# Hormonal abnormalities in alexithymia

**DOI:** 10.3389/fpsyt.2022.1070066

**Published:** 2023-01-09

**Authors:** Katharina S. Goerlich, Mikhail Votinov

**Affiliations:** ^1^Cognitive Neuroscience Center, Department of Biomedical Sciences of Cells and Systems, University Medical Center Groningen, University of Groningen, Groningen, Netherlands; ^2^Institute of Neuroscience and Medicine 10, Research Centre Jülich, Jülich, Germany; ^3^Department of Psychiatry, Psychotherapy and Psychosomatics, Medical Faculty, RWTH Aachen University, Aachen, Germany

**Keywords:** alexithymia, social, emotional, cortisol, oxytocin, vasopressin, thyroid hormones, sex hormones

## Abstract

Alexithymia is a personality trait characterized by difficulties in emotion recognition and regulation that is associated with deficits in social cognition. High alexithymia levels are considered a transdiagnostic risk factor for a range of psychiatric and medical conditions, including depression, anxiety, and autism. Hormones are known to affect social–emotional cognition and behavior in humans, including the neuropeptides oxytocin and vasopressin, the steroid hormones testosterone and estradiol, the stress hormone cortisol as well as thyroid hormones. However, few studies have investigated hormonal effects on alexithymia and on alexithymia-related impairments in emotion regulation and reactivity, stress response, and social cognition. Here, we provide a brief overview of the evidence linking alexithymia to abnormalities in hormone levels, particularly with regard to cortisol and oxytocin, for which most evidence exists, and to thyroid hormones. We address the current lack of research on the influence of sex hormones on alexithymia and alexithymia-related deficits, and lastly provide future directions for research on associations between hormonal abnormalities and deficits in emotion regulation and social cognition associated with alexithymia.

## 1. Introduction

Alexithymia is a personality construct characterized by difficulty understanding one’s own emotions, distinguishing them from bodily signals of arousal, and verbalizing one’s feelings to others. The term “alexithymic” was coined by Sifneos (1) to describe a variety of patients with psychosomatic diseases who showed a common deficit, a marked difficulty finding words to describe their feelings. The alexithymia concept captured the attention of specialists in psychosomatic medicine and became the main theme of the 11th European Conference on Psychosomatic Research (2). Despite several debates, alexithymia was conceptualized as a hypothetical construct that could be operationalized and empirically evaluated [for reviews on the history and debates surrounding the alexithymia construct, see (3, 4)]. While several theoretical models of the alexithymia construct exist (5), the dominant model encompasses (1) difficulty in identifying and describing feelings; (2) difficulty in distinguishing between feelings and bodily sensations of arousal; (3) restrained and limited imaginative processes; and (4) a cognitive style oriented toward the outside (6, 7). As a consequence of these difficulties, alexithymia is associated with reduced empathy for others, impulsive behaviors, and problems in interpersonal relationships, and individuals with high degrees of alexithymia are sometimes described as being unimaginative and boring, having a stiff and wooden posture, and to seem cold and distant in social interactions [e.g., (8, 9)].

Today, most researchers agree that alexithymia is a dimensional, normally distributed, subclinical personality trait [e.g., (4, 10, 11)]. While several methods exist to assess alexithymia, both interviews and self-report scales, the most widely used measure is the Toronto Alexithymia Scale [TAS-20; (12)]. The TAS-20 is a five-point Likert-type 20-item self-report scale that assesses the total alexithymia level as well as three factors: difficulties identifying feelings (DIF), difficulties describing feelings (DDF), and externally oriented thinking (EOT). The international cut-off for clinically relevant alexithymia is a TAS-20 total score > 61, based on which an alexithymia prevalence of 10% in the general population was observed (13, 14).

Alexithymia is considered a transdiagnostic risk factor for affective disorders (e.g., depression, anxiety, and post-traumatic stress disorder). Moreover, alexithymia is associated with medical conditions that include a psychosomatic component (i.e., pain syndromes, gastroenterological, and dermatological disease), but also with primarily medical conditions such as cancer, cardiological, and neurological diseases. Theories linking alexithymia and physical illness involve the physiological level [e.g., the hypothalamic-pituitary-adrenal (HPA) axis, chronic sympathetic hyperarousal, inflammation], but also the behavioral and the cognitive level [e.g., illness perception and behavior, somatic amplification; for recent reviews, see (15, 16)]. Despite five decades of research, the intricate relationship between alexithymia and mental and physical disease proneness is still far from being fully understood.

In 1985, the stress-alexithymia hypothesis was proposed, stating that alexithymic individuals have difficulties identifying the emotions that are linked to physiological arousal when experiencing a stressful situation (17). These difficulties are accompanied by increased somatic markers (e.g., increased heart rate and higher electrodermal activity), resulting in sustained autonomic hyperactivity, which in turn may lead to psychologically and physiologically harmful consequences. In their review, Panayiotou et al. (16) found that several studies reported hyperarousal at rest in alexithymia or during emotional stimulation, which may indicate poor regulation of emotional response intensity. However, many studies observed opposing findings of attenuated autonomic responses, indicating diminished automatic emotion processing or aberrant emotion regulation involving blunted emotional responses, emotion suppression, or avoidance.

A further important hypothesis to understand the relationship between alexithymia and altered physiological response is the decoupling hypothesis (18). This hypothesis suggests a dissociation between physiological arousal and subjective awareness of emotions in alexithymic individuals. A recent review identified decoupling between different emotion response systems as a consistent finding in the alexithymia literature, concluding that “the large majority of studies support the presence of decoupling in alexithymia, with evidence clearly in the direction of blunted physiological activation and normal or heightened reported affect” (16).

Despite the large body of literature on the physiological, behavioral and cognitive levels of alexithymia, relatively few studies investigated the role of potential hormonal imbalances contributing to alexithymia. Hormones such as oxytocin and cortisol are well known to affect emotion regulation and social cognition, which represent areas of major difficulty for individuals with high alexithymia levels, who struggle recognizing their own feelings and those of others, show reduced empathy and less altruistic behavior, and experience less distress watching others in pain [e.g., (19, 20); for a review on alexithymia and theory of mind, see (21); for a review on alexithymia and empathy, see (22)]. In the last two decades, the influence of hormones on human social–emotional behavior has received increasing scientific attention. A review on single hormone administration studies established the important roles of the neuropeptides oxytocin and vasopressin as well as of the steroid hormones testosterone and estradiol on social–emotional behavior in humans (23).

Hormones and stress are inherently linked: A stressful experience can trigger a cascade of hormonal responses. Upon receiving a distress signal from the amygdala, the hypothalamus activates the sympathetic nervous system and the HPA axis, prompting enhanced secretion of glucocorticoids such as cortisol, catecholamines, growth hormone, and prolactin, which results in a burst of energy, a phenomenon known as the “fight-or-flight” response. The stress response is necessary to maintain homeostasis, but long-term activation of the stress system can be hazardous or even lethal (24). Affective disorders such as depression and anxiety are associated with abnormalities in the stress system [for a review, see (25)]: Stress promotes the occurrence of mood disorders, and these pathologies are related to modifications of HPA axis functioning (26). Psychosocial stress in childhood can program the HPA axis permanently into hyperactivity (27), rendering individuals vulnerable to environmental stress that can further activate the HPA axis (25). The stress-alexithymia hypothesis suggests the existence of a “viscous circle” between vulnerability to environmental stress in individuals with high alexithymia levels and failure to down-regulate emotional arousal, resulting in sustained autonomic hyperarousal. Indeed, some studies provided evidence for altered HPA axis functioning in alexithymia [for a review, see (15)].

In sum, alexithymia is a transdiagnostic risk factor for conditions such as depression, anxiety, and autism, all of which have been linked to hormonal abnormalities. Yet, very few studies have investigated associations between hormones and alexithymia. Here, we provide an overview of evidence on hormonal abnormalities in alexithymia, and explain why in our opinion future research would benefit from more thorough investigations into hormonal contributions to alexithymia and deficits in emotion regulation and social cognition as well altered physiological responses to stress associated with this personality trait.

## 2. Hormone studies in alexithymia

### 2.1. Cortisol

Cortisol, known as the stress hormone, is a glucocorticoid hormone produced in the adrenal gland. Studies demonstrated that cortisol promotes cognitive control of negative emotions (28, 29), that cortisol administration reduced phobic fears (30, 31) and that cortisol levels correlated with emotionality (32).

A potential association between the stress hormone cortisol and alexithymia was already tested in the early 90s (33). Based on observations that alexithymia can result from traumatic stress and on findings of high norepinephrine/cortisol ratios in post-traumatic stress disorder (PTSD) [for a review on the effects of traumatic stress on the brain, see (34)], Henry et al. (33) correlated norepinephrine and cortisol levels with alexithymia levels in a small group (*N* = 17) of formerly alcohol-dependent men. Norepinephrine/cortisol ratios correlated significantly with alexithymia, reminiscent of observations in PTSD. The authors suggested that higher alexithymia levels may be accompanied by an increasing functional separation between the sympatho-adrenal medullary axis and the HPA axis and a failure of the HPA axis to respond properly.

Härtwig et al. (35) investigated the cortisol awakening response (CAR; the rapid increase of cortisol levels directly after waking up in the morning) in individuals with low (*N* = 37) versus high (*N* = 41) alexithymia (35). The CAR measures basal activity of the HPA system, the stress hormone system that regulates stress responses and adaptation to environmental challenges. Chronic hyperactivation of the HPA-system is common in stress-related disorders (36). Härtwig et al. (35) found that high alexithymics had a significantly lower mean-CAR than low alexithymics, a result that remained stable after controlling for age and biological sex. Healthy individuals with high alexithymia levels thus seem to exhibit hypoactivity of the HPA system as measured by CAR. In addition, compared to low-alexithymics, high-alexithymics reported to experience higher levels of stress in interpersonal interactions and showed more extreme decoupling between physiological response and psychological perception.

Cortisol awakening response was also tested in female patients with bulimia nervosa with and without alexithymia (37). A significant reduction in CAR magnitude was observed in alexithymic compared to non-alexithymic patients, in line with the previous finding in healthy individuals. However, this study differentiated between the overall production of cortisol, measuring HPA basal activity, and overall cortisol increase, measuring HPA sensitivity. HPA basal activity, but not sensitivity, was decreased in alexithymic compared to non-alexithymic patients. This suggests intact HPA axis sensitivity, but reduced HPA basal activity, which may result in inadequate cortisol production and thus impaired stress response in alexithymia. However, an earlier study in patients with somatoform disorders found no association between alexithymia and HPA basal activity and sensitivity (38).

Kano et al. (39) investigated the effects of corticotrophin-releasing hormone (CRH), the central driver of the HPA axis, on adrenocorticotropic hormone (ACTH) responses in relation to alexithymia in patients with irritable bowel syndrome compared to healthy controls. Across the sample, individuals with higher alexithymia levels showed significantly stronger ACTH responses to CRH injection, demonstrating a stronger endocrine stress response to CRH with higher alexithymia.

In sum, studies on cortisol suggest that alexithymia is linked to an altered function of the HPA axis, specifically reduced HPA basal activity, indexed by higher norepinephrine/cortisol ratios, reduced CAR, and an exaggerated ACTH stress response to CRH.

### 2.2. Thyroid hormones

Thyroid hormones play essential roles in mood regulation and cognition. The thyroid-stimulating hormone (TSH) is a hormone released by the pituitary gland. TSH stimulates the thyroid gland to produce thyroxine (T4), which regulates metabolism, mood, and body temperature. T4 is converted to triiodothyronine (T3), which is the active hormone that stimulates metabolism and oversees bone health. Free triiodothyronine (FT3) is elevated in hyperthyroidism and decreased in hypothyroidism. Autoimmune thyroiditis is linked to depression and anxiety disorders [for a systematic review and meta-analysis, see (40–43)].

Only one study so far investigated the relationship between thyroid hormone levels and alexithymia, albeit in a postpartum setting (44). The authors measured serum thyrotropin (TSH), free thyroxine (FT4) and free triiodothyronine (FT3) hormone levels, and TAS-20 alexithymia levels in 74 healthy women on day 3 postpartum. Alexithymic versus non-alexithymic women were found to differ significantly in their thyroid hormone levels: The former had significantly lower FT4, higher FT3, and lower FT4:FT3 ratios than the latter, indicative of altered thyroid homeostasis in alexithymia. These relationships remained stable after controlling for postpartum depression. These findings suggest that alexithymia is linked to an imbalance between the amount of thyroid hormones needed by the body and the amount of thyroid hormones available, providing initial evidence for a link between alexithymia and thyroid dysfunction.

In a recent pilot study, Martino et al. (45) investigated the associations between alexithymia and depression, anxiety, and health-related quality of life in patients with Hashimoto’s thyroiditis. Patients with Hashimoto’s and control patients with general thyroid disease participated in this study. Across the sample, the majority (89.2%) of patients were alexithymic (TAS-20 score > 60) or potentially alexithymic (TAS-20 score 52–60). Although patients with Hashimoto’s also had mild depression and moderate to severe anxiety, TAS-20 sum scores did not correlate with the other scores in patients with Hashimoto’s (only DDF showed a moderate, inverse correlation with the cognitive component of depression). These results indicated an association between alexithymia and Hashimoto’s thyroiditis that was largely independent from comorbid anxiety and depression.

Hasegawa et al. (46) examined TAS-20 alexithymia scores in surgical patients with thyroid disease (Graves’ disease and papillary thyroid cancer) before and after thyroidectomy surgery. After surgery, all patients scored significantly higher on the DIF factor of the TAS-20 alexithymia scale, compared to pre-surgically. Although hormone levels indicative of thyroid function were not assessed in this study, these results suggest an increase in DIF alexithymia levels following thyroid removal. However, the authors provided no physiological mechanism potentially underlying these changes, and no follow-up was performed, leaving potential long-term effects unknown.

In a further study, an alexithymia prevalence of 51.9% in 162 patients with chronic autoimmune thyroiditis was reported (47). The authors suggested that “alexithymia is not only an indirect risk factor of the autoimmune thyroiditis development but also a predictor of the disease course.”

In sum, very few studies examined potential associations of alexithymia with thyroid hormones, providing initial evidence for lower FT4, higher FT3, and lower FT4:FT3 ratios in alexithymic individuals, suggesting thyroid dysfunction. In addition, patients with chronic thyroid disease showed a much higher alexithymia prevalence (51.9%) than in the normal population (10%), and patients with thyroid disease had significantly more DIF after thyroidectomy than before surgery.

### 2.3. Oxytocin and vasopressin

Oxytocin and Arginine Vasopressin (AVP) are peptide hormones synthesized in the hypothalamus. Across different mammalian species, including humans, it has been demonstrated that AVP regulates social behaviors such as affiliation (48), social recognition (49), and cooperative behavior (50). In addition, AVP administration enhanced encoding of social information (51, 52), and AVP deficiency in patients is linked to deficits in categorizing socio-affective stimuli (53).

Many studies investigated the role of oxytocin on emotion regulation and social cognition. Intranasal oxytocin application increased positive communication during interpersonal conflict (54), modulated visual attention toward social signals of positive approach and threat (55–57), and improved social cognition (58–60). In fact, oxytocin administration has been suggested as a treatment for a number of disorders related to emotional and social dysfunction, including depression, anxiety, and autism [for reviews, see (49, 61)].

Luminet et al. (62) investigated the effects of intranasal oxytocin administration on individuals of varying socio-emotional ability in a randomized controlled trial including 60 male students who either received oxytocin or a placebo before performing the Reading the Mind in the Eyes test. The performance of individuals with lower alexithymia levels was equally good in both oxytocin and placebo conditions, whereas the performance of individuals with high alexithymia levels improved under oxytocin compared to placebo. These results demonstrated that oxytocin can improve socio-emotional competence in individuals with high alexithymia levels.

Koh et al. (63) hypothesized that variations in the oxytocin receptor gene might contribute to social-emotional difficulties in alexithymia. They tested potential associations between oxytocin receptor gene single nucleotide polymorphisms or haplotypes and alexithymia in 355 patients with obsessive-compulsive disorder. Single-marker and haplotype association analyses were performed with eight single nucleotide polymorphisms. However, no significant associations could be detected, leaving a role of genetic variations in the oxytocin receptor gene in alexithymia unclear.

Schneider-Hassloff et al. (64) focused on the single nucleotide polymorphism rs53576 in the oxytocin receptor gene and its interaction with childhood attachment security in 195 healthy participants. A subgroup (*N* = 163) additionally underwent functional magnetic resonance imaging (fMRI), during which they played the prisoner’s dilemma game, an interactive mentalizing task. It was observed that rs53576 and childhood attachment security jointly modulated alexithymia levels. Specifically, alexithymia levels were significantly higher in rs53576 GG-homozygotes with insecure versus secure childhood attachment. Moreover, lower alexithymia levels were associated with greater volumes of the superior parietal lobule, part of the parieto-frontal human mirror system (65) that is important for mentalizing abilities. These findings suggest that a gene-environment interaction between the rs53576 variation in the oxytocin receptor gene and insecure childhood attachment contributes to deficits in emotional awareness, the core characteristic of alexithymia.

Baskaran et al. (66) correlated serum oxytocin levels obtained every 5 min over a period of 10 h with alexithymia levels (amongst other measures) in healthy men. They observed strong inverse correlations between TAS-20 alexithymia levels and the secretory dynamics of oxytocin, which were driven by DIF. Additionally, subjects with reduced pulsatile release of oxytocin showed a more avoidant attachment style and felt less supported. These results further corroborate the role of oxytocin as a key mediator of social-emotional functioning and provide additional evidence for impaired oxytocin release patterns in high-alexithymic individuals.

Another study tested the relationship between oxytocin levels and social-emotional functioning in 79 women with anorexia nervosa (67). TAS-20 sum scores and DIF correlated negatively with oxytocin levels across the whole sample. These relationships remained significant after controlling for body-mass index and estrogen levels, providing further evidence for low oxytocin levels contributing to deficits in social-emotional functioning associated with alexithymia.

Recently, the relationship between alexithymia and oxytocin was investigated in perpetrators of intimate partner violence and non-violent controls (68). Effects of an empathic induction task (videos evoking negative emotions) were tested on endogenous salivary oxytocin levels, mood state, and emotional perception. Perpetrators of intimate partner violence were found to be significantly more alexithymic than controls. Importantly, salivary oxytocin levels were linked to a greater mood state response, a more intense perception of the video character’s emotional state, and lower alexithymia levels across the whole sample. These findings suggest that in addition to lower alexithymia levels, higher oxytocin levels are associated with a greater understanding of one’s own emotions, a greater insight into the emotional state of others, and a more accurate emotional response to others emotional states.

In sum, several studies on oxytocin showed that alexithymia is linked to reduced oxytocin levels. Further, there is evidence for a gene-environment interaction contributing to alexithymia, with variation in the oxytocin receptor gene and insecure childhood attachment contributing to high alexithymia levels. Notably, a randomized controlled trial demonstrated that intranasal oxytocin administration can improve social-emotional functioning in high-alexithymic individuals. Despite these promising results and the known beneficial effects of oxytocin on social cognition and communication [e.g., (54, 58)], there have been no further investigations on potentially beneficial effects of oxytocin on social cognition problems or reduced empathy and mentalizing abilities associated with alexithymia.

### 2.4. Sex hormones

Besides the hormones discussed above, the sex hormones testosterone, progesterone, and estradiol have been associated with social cognition and emotion processing, abilities that are impaired in alexithymia. Testosterone administration, for instance, influenced anger levels (69, 70), was associated with changes in connectivity within the emotion regulation network (53, 71), and enhanced empathic concern in men (72). In women, testosterone application affected emotional functions (73), enhanced responsiveness to social threat (74), and diminished cognitive empathy (75). Moreover, Machiavellian trait patterns [which are significantly associated with alexithymia; e.g., (76)] interacted with the application of testosterone and AVP and modulated aggression levels (77).

In addition, changes in estradiol and progesterone levels during menstrual cycle phases are associated with emotion recognition accuracy and emotional memories [for a review, see review (78)], emotion recognition and perspective taking performance (79–81), and fear recognition (82). Moreover, estradiol application modulated emotion regulation in women (83), and resulted in an increase in emotional reactivity in men (73). Five decades of research have demonstrated that alexithymia is linked to deficits in emotion recognition and regulation, emotional memory and perspective taking (84), yet differences in the sex hormones testosterone, progesterone, and estradiol in relation to alexithymia have received little scientific attention.

A study on 84 infertile men reported that alexithymia was negatively correlated with stress hormones but not with sex steroids (85). In climacteric (menopausal) women, alexithymia was associated with significantly higher estradiol levels (86). A further study observed that higher testosterone levels in combination with lower alexithymia levels predicted higher sexual desire in women (87), but direct relationships between alexithymia and testosterone levels were not reported. These findings provide initial evidence for an association between alexithymia and the sex hormones testosterone and estradiol, but much more research is needed to shed light on this potential link.

## 3. Conclusion and future directions

Alexithymia is associated with impaired stress tolerance and reduced emotion regulation abilities. Therefore, studies on its association with hormonal abnormalities are of particular importance. Several studies focused on the role of the HPA axis in relation to alexithymia, a key mediator of physiological and psychological stress responses. Hormones also represent a link between psychological stress and alterations of the immune system, given that acute and chronic stress can impair cellular stress responses and pro-inflammatory cytokines while activating anti-inflammatory agents of the immune system (15).

The existing evidence suggests that there is a significant link between alexithymia and differences in cortisol levels, evidenced by higher norepinephrine/cortisol ratios (reminiscent of those observed in PTSD), lower CAR, and stronger endocrine stress responses to corticotrophin-releasing hormone (CRH). These results suggest that alexithymia is indeed linked to an altered function of the HPA axis. HPA axis functioning is influenced by factors such as age, sex, and hereditary predisposition, in combination with early childhood experiences and personality characteristics (88), factors that are also associated with alexithymia (89). Since the HPA axis is a key mediator of psychological and physiological stress responses, more studies are needed to investigate the association between alexithymia and altered HPA axis functioning more thoroughly to find out whether this association represents a direct link or an indirect link *via* other factors. Future studies could apply stress tests in combination with cortisol measures to shed light on the associations between alexithymia and cortisol levels, ACTH, and CRH within the context of responses to acute stress.

Thyroid diseases are linked to mood disorders such as anxiety and depression, yet very little research has been devoted to investigating the relationship between alexithymia and thyroid hormones and thyroid diseases. The existing evidence suggests that alexithymia is indeed associated with altered thyroid hormone levels, revealing a pattern of lower FT4 and higher FT3, indicative of thyroid dysfunction. Such a pattern of lower FT4 and higher FT3 was also identified in a randomized controlled trial to be related to the recurrence of depressive episodes in patients with bipolar disorder (90), further corroborating the link between abnormalities in thyroid functioning and mood disorders. Moreover, there is initial evidence for a high prevalence of alexithymia in thyroid diseases as well as for significant changes in alexithymia after thyroidectomy. Given this sparse, but significant evidence, we think that it would be worthwhile to conduct future studies on the link between thyroid abnormalities and alexithymia. Thyroid hormones and sex hormones could be measured in combination with alexithymia levels in healthy individuals and in patients with thyroid and other autoimmune diseases to better understand the complex associations between alexithymia, hormonal abnormalities, and alterations of the immune system.

Several studies focused on the role of oxytocin, demonstrating that alexithymia is linked to reduced oxytocin levels and that intranasal oxytocin administration can improve socio-emotional performance specifically in high-alexithymic individuals. Unfortunately, no further studies have tested potentially beneficial effects of oxytocin on alexithymia-related problems such as difficulties in social cognition or reduced empathy. Also surprising is the fact that there are currently no systematic investigations into associations between alexithymia and AVP vasopressin, which is–like oxytocin–known to play an important role in social cognition and behavior [for reviews, see (25, 91)]. AVP strongly contributes to the endocrine and neural response to stress, and affective disorders seem to be related to excessive vasopressin function [for a review, see (26)]. However, to our knowledge, only two studies on AVP included alexithymia as a variable, but reported no analysis results on associations between AVP and alexithymia (92, 93). As concluded in Section “2.1 Cortisol,” alexithymia is related to an exaggerated ACTH stress response to CRH. AVP has been shown to strongly potentiate ACTH-releasing activity, and in acute stress, CRH is the main cause of increased ACTH release, whereas in chronic stress, there seems to be a switch from CRH to AVP stimulation of ACTH release (25). Given these relationships, we think that future investigations into potential effects of AVP on ACTH stress response to CRH in relation to alexithymia could be informative. Hormone administration studies could be conducted to investigate the effects of oxytocin and AVP vasopressin on alexithymia-related impairments in stress response, emotion regulation, and social cognition.

On a more general note, future research would benefit from testing links between alexithymia and hormonal imbalance in big data sets rather than small samples using correlational approaches. Age and biological sex should always be taken into account in such studies. Machine learning and unbiased cluster analyses could be performed in healthy individuals and in various disorders to test, for example, links between alexithymia and variations in cortisol and oxytocin receptor genes.

In conclusion, while the topic is still in the infant stage, there is accumulating evidence for significant links between alexithymia and hormonal dysregulation. Therefore, we consider it important to continue this research line to extend our understanding of the role alexithymia plays in the stress-hormone system. Future studies should investigate the mechanisms underlying alexithymia and individual vulnerability to stress, and the extent to which individual differences in alexithymia levels mediate susceptibility to affective disorders *via* hormonal abnormalities and the stress-hormone system.

## Data availability statement

The original contributions presented in this study are included in the article/supplementary material, further inquiries can be directed to the corresponding author.

## Author contributions

KG conceptualized the manuscript. KG and MV wrote sections of the manuscript and provided the first draft, contributed to the manuscript revision, read, and approved the submitted version.
